# Evaluation of errors and limits of the 63-μm house-dust-fraction method, a surrogate to predict hidden moisture damage

**DOI:** 10.1186/1756-0500-2-218

**Published:** 2009-10-24

**Authors:** Christoph Baudisch, Ojan Assadian, Axel Kramer

**Affiliations:** 1 State Health and Social Office of Mecklenburg-Pomerania, Branch Office Schwerin, Germany; 2 Clinical Institute for Hygiene and Medical Microbiology of the Medical University of Vienna, Department of Hospital Hygiene, Vienna General Hospital, Waehringer Guertel 18-20, 1090 Vienna, Austria; 3 Institute for Hygiene and Environmental Medicine, Ernst Moritz Arndt University, Greifswald, Germany

## Abstract

**Background:**

The aim of this study is to analyze possible random and systematic measurement errors and to detect methodological limits of the previously established method.

**Findings:**

To examine the distribution of *random errors *(repeatability standard deviation) of the detection procedure, collective samples were taken from two uncontaminated rooms using a sampling vacuum cleaner, and 10 sub-samples each were examined with 3 parallel cultivation plates (DG18). In this two collective samples of new dust, the total counts of *Aspergillus spp*. varied moderately by 25 and 29% (both 9 cfu per plate). At an average of 28 cfu/plate, the total number varied only by 13%.

For the evaluation of the influence of old dust, old and fresh dust samples were examined. In both cases with old dust, the old dust influenced the results indicating false positive results, where hidden moist was indicated but was not present. To quantify the influence of sand and sieving, 13 sites were sampled in parallel using the 63-μm- and total dust collection approaches. Sieving to 63-μm resulted in a more then 10-fold enrichment, due to the different quantity of inert sand in each total dust sample.

**Conclusion:**

The major errors during the quantitative evaluation from house dust samples for mould fungi as reference values for assessment resulted from missing filtration, contamination with old dust and the massive influence of soil. If the assessment is guided by indicator genera, the percentage standard deviation lies in a moderate range.

## Background

Quantitative measurements of mould colonizing in the 63-μm fraction of house dust is a suitable surrogate method to evaluate present or hidden moisture damage [1]. As there always will be an influence by the outdoor air, we assessed methodological possibilities to control this influence at the level of interpretation of the measurements. The further development of the assessment model was based on two facts influenced by the outdoor air: first, the background load of all species increases in summer. This is compensated through a percent evaluation of moulds relative to the total number. Second, the indicator genera *Aspergillus spp*., *Eurotium spp*., or *Penicillium spp*. occur "relatively" constantly throughout the year in rooms. Hence, the increased amounts of these genera indicate moisture damage. The percent evaluation of indicator genera is intended - above a base load in winter - to eliminate the summer influence (Table 1).

**Table 1:** House dust evaluation for mould in the 63-μm fraction, modified one-step assessment model

**Corresponding total count without**	**concentration limit, indicator genus**	
yeast (cfu/g)	*Aspergillus- + Eurotium spp *(cfu/g) or proportion (%)	*Penicillium spp *(cfu/g) or proportion (%)
0 - 500,000	≥ 200,000 cfu/g	≥ 300,000 cfu/g
500,000 - 2,000,000	≥ 20%	≥ 20%
> 2,000,000	≥ 420,000 cfu/g	≥ 450,000 cfu/g

Our previous measurements allowed generation of reference values for a house dust monitoring method to detect hidden moisture damage controlling widely for the influence of outdoor air, accumulated old dust, and dust swirled up from room surfaces. The aim of the present study was to analyze possible random and systematic measurement errors and to detect methodological limits of the previously established method caused by statistical broad standard deviations, influence of old dust, sand and sieving, contamination with soil, stability of samples, mass or surface relation, assessment by indicator genera, sampling with filter holder or with vacuum-cleaner.

## Findings

### Random errors

To evaluate the distribution of *random errors *(repeatability occurring standard deviation) of the measurement, a collective sample was taken from a non-colonized (non-mouldy) room using a sampling vacuum-cleaner (Vampirette, Siemens, Germany), and 10 sub-samples each were examined with 3 parallel plates (DG18) from the filtered 63-μm dust fraction. Rooms were defined as non-colonized if no moisture damage was visible and no plants or caged animals were present. In this room with low concentrations (average total colony count 17,900 cfu/g), the relative standard deviation for *Aspergillus spp*. was moderate (25%) with an average of 9 cfu/plate. Below 4 cfu/plate, the relative standard deviation markedly increased (between 60 and 100%), and was above 100% at < 1 cfu/plate (Table 2). In another non-colonized (non-mouldy) room with higher concentrations (average total count 280,000 cfu/g) and averages of 3-9 cfu/plate for all yielded genus, the relative standard deviations varied between 29 and 39%. At an average of 28 cfu/plate, the total count varied only by 13% (Table 3).

**Table 2:** Standard deviations of 10 sub-samples (3 parallel examinations) of a house-dust sample with low load in the 63-μm fraction in cfu/g × 1,000 (DG18, 25°C).

**Number**	**1**	**2**	**3**	**4**	**5**	**6**	**7**	**8**	**9**	**10**	**Mean**	**% TCC**	**SD**	**SD%**
Asp.	12	13	8	8	9	9	6	11	7	8	9.1	51	2.2	25

Peni.	6	3	3	2	5	1	2	5	1	7	3.5	20	2.1	61

Clad.	5	0	2	1	5	5	1	0	3	1	2.3	13	2.1	89

Wall.	2	0	0	1	0	2	1	0	0	0	0.6	3	0.8	141

Aureo. pu.	0	0	0	0	0	1	0	0	0	0	0.1	1	0.3	316

Muc.	0	0	1	0	0	0	0	0	0	0	0.1	1	0.3	316

Fusa.	0	0	0	0	0	0	1	0	0	0	0.1	1	0.3	316

Myc. st.	1	3	4	3	1	6	2	0	0	1	2.1	12	1.9	91

TCC	26	19	18	15	20	24	13	16	11	17	17.9	100	4.6	26

SD- Standard deviation, SD% - relative percent standard deviation, % TCC- percent proportion of total colony count

**Table 3:** Standard deviations of 10 sub-samples (3 parallel examinations) of a house-dust sample in the 63-μm fraction in cfu/g × 10,000 (DG18, 25°C).

**Number**	**1**	**2**	**3**	**4**	**5**	**6**	**7**	**8**	**9**	**10**	**Mean**	**% TCC**	**SD**	**SD%**
Aspergillus	12	7	8	8	7	10	15	11	8	6	9	34	3	29

Penicillium	5	4	5	2	4	3	3	7	4	4	4	15	2	35

Cladosporium	10	6	8	6	7	8	5	5	6	7	7	24	2	24

Alternaria	2	5	3	2	4	3	3	3	2	2	3	10	1	39

Other	5	4	3	4	3	4	4	3	5	9	5	16	2	36

TCC	35	26	28	21	25	28	29	30	25	28	28	100	4	13

SD- Standard deviation, SD% - relative percent standard deviation, % TCC- percent proportion of total colony count

These results indicate that the higher the contamination of rooms is with mould fungi, the more reliable the detection method becomes. For measurements of the background concentration (Table 2), the method quickly reaches its detection limits because of low concentrations and very high standard deviations, which however, have no implication for measurements within the concentrations limits. According to ISO/DIS 16000-17:2006 and Gabrio T et al. [2,3], the best results are achieved if more than 10 cfu per plate and less than 100 cfu per plate (90 mm diameter) are yielded. Generally, the best yield ranges within 20 to 40 cfu per plate, a range where the standard deviation of the results is at a minimum. Indeed, as shown in table 1, our results for optimal assessment of moulds in the environment range between 200,000 to 450,000 cfu per gram dust, corresponding to 20 to 45 cfu per plate in a dilution of 1:10,000, result in the lowest standard deviations. By counting species as indicator genera, the total count, together, the random error diminishes.

### Old Dust

To investigate potential problems with old dust, dust samples were vacuumed from the carpet (fresh dust, 2 weeks old) and from top of a closet (old dust, 1 year old) in a bedroom. The fresh dust showed no physical enrichment (*Aspergillus spp*. and *Eurotium spp*. = 23,000 cfu/g, *Penicillium spp*. = 33,000 cfu/g). In contrast, the indicator genera in the old dust exceeded concentration limits according to table 1 (measurement 500,000 and 700,000 cfu/g). The indicator genera *Aspergillus spp*. + *Eurotium spp*., and *Penicillium spp*. were each enriched by the factor of 21. Therefore, the sample of old dust would have to be classified as contaminated, and does not represent the true and actual condition of the environment.

Guideline VDI 4300 Part 8, 2001 [4] distinguishes between fresh (definite age, according to our method one week) and old dust (indefinite age). Generally, old dust contains much higher fungal concentrations, because it has been enriched longer and is usually much finer in particle size. The smaller the particle size of dust, the higher is the expected enrichment phenomenon. For instance, old dust results from airborne dust of particle size < 10 μm settles and accumulates on cupboards and closets or under beds without influence of dilution with other particles. Therefore, our method allows sampling of accessible floors and surfaces.

In a dry area the previously not attainable old dust under a cabinet together with fresh dust was absorbed and investigated. The amount of total count indicated false positive dampness damage (totally count 521,700 cfu/g, 74% *Penicillium spp*.). After repeating the measurement using only fresh dust a correct result expectable for a definitive dry area was measured (total count 62,500 cfu/g, 16% sum of indicator genera) [5]. Therefore, inaccurate findings will result by sampling old dust and assessment of the values according to table 1.

### Influence of sand and sieving

In order to investigate the influence of sieving, table 4 compares the total counts of mould spores per gram of dust from one sample each of vacuumed house dust in the sieved 63-μm fraction and in non-sieved total dust (cultivation of each sample on three DG 18 agar plates, see [1]). Sieving to 63-μm lead to a greater than 10-fold enrichment due to the different quantity of inert sand in each total dust sample.

**Table 4:** Mould spore concentration (total counts) per g house dust of the 63-μm fraction vs. the total dust fraction

**No.**	**63-μm dust (cfu/g)**	**Total dust (cfu/g)**	**Factor**	**Remark**
1	156,500	156,000	1.0	Living-room

2	616,000	554,000	1.1	Bedroom

3	2,250,000	1,450,000	1.5	Home

4	97,000	60,000	1.6	Living-room

5	82,000	39,000	2.1	Office

6	272,000	116,000	2.3	Living-room

7	93,000	33,000	2.8	Office

8	120,000	31,000	3.9	Office

9	683,000	115,000	5.9	Living-room

10	71,000	10,000	7.1	Office

11	360,000	44,500	8.1	Office

12	833,000	78,000	10.7	Living-room

13	1,366,000	93,000	14.7	Attic

In case 1 (table 4), the total dust corresponded to the 63-μm dust (no sand in the house). This house was vacuumed daily. The dust contained 88,500 cfu/g *Aspergillus spp*. + *Penicillium spp*., and without sieving would have been classified as a contaminated home, assessed after Schleibinger et al. [6]. According to our assessment, the sample was conclusively without mould, which in this case correlated with the inspected location. The indicator genera concentrations were 5 times below the cut-off value (table 1). Schleibinger et al. [6] found a specificity of 98%, which is comparable to our results, and a sensitivity of 93%, without sieving in 47 non-contaminated and 43 contaminated homes, although the assessment was performed "only" according to indicator genera and the moisture damage was clearly visible. We achieved a sensitivity of 100% for the house-dust method with DG18. It is likely that the comparatively poor sensitivity in contaminated homes [6] is the result of a higher influence of contamination with sand, which leads to a dilution of the samples. Therefore, sieving the house dust to the 63-μm fraction is considered imperative for assessing moisture damage.

Sieving corresponds to geological preparation of soil samples [7,8]. Through sieving, quantitatively comparable reference values are obtained, i.e., in total dust samples, the influence of sand is responsible for results that differ by a factor of up to 10 (+ 900% error). However, even small differences of a factor of 2 or 3 result in errors of +100 to +200%. Such effects are also to be expected for chemical analysis, mycotoxins or quantitative polymerase chain reaction. However, sieving results the reference to exposure to be lost (see uptake via hand-to-mouth contact). Therefore, every task requires its own measurement strategy.

### Soil

The influence of soil was seen in the analysis of house-dust samples from a location where shoes were changed in the entry hall of an inhabited garden cottage (total count 486,057 cfu/g, *Aspergillus spp*. and *Eurotium spp*. 327,384 cfu/g) and from a sample from a school classroom (total count 516,666 *Aspergillus spp*. and *Eurotium spp*. 43%). Neither sample was taken from a living area. The classroom floor was not carpeted. In contrast, the influence of soil in homes was negligible [9].

### Stability of samples

The protocol for house-dust analyses stipulates that inoculation and incubation of samples be performed in the laboratory at the latest the day after sampling. But in actual practice, the question must be asked whether interim storage of the dry house dust (e.g., during shipping) can result in errors. In a house-dust sample without moisture damage (sampling on 04.09.06; total count 623,000 cfu/g, *Aspergillus *spp. and *Eurotium *spp. 103,000 cfu/g, *Penicillium *spp. 16,700 cfu/g), the counts of the genera *Aspergillus *spp. and *Eurotium *spp., and *Penicillium *spp. remained constant over 3 weeks at room temperature (persistence of indicator genera). In contrast, the amount of *Cladosporium *spp. decreased by 72% (-30% total count per 2 weeks). Koch et al. [10] found a decreasing frequency of 10% for the total count over 2 weeks at a storage temperature of 4°C. Here, too, *Cladosporium *spp. was the limiting factor. Still lower storage temperatures should further reduce the rate of decrease [10]. It is apparent that through storage, the total assessment is only influenced by the total count, so that after 1 week of storage at 4°C, an erroneous assessment is not likely to result. Nevertheless, interim storage times should be kept to a minimum, or a reduction in total count should be taken into consideration.

### Indicator genera

The exclusive evaluation according to indicator genera inevitably leads to fewer false-positive findings than does an evaluation method with more species and genera. *Eurotium *spp. is an additional indicator of moisture damage, which, compared to other moisture indicators, occurs frequently. However, it is problematic that the additional moisture assessment parameters - as opposed to the indicator genera - according to [9] often scored levels of zero. Thus, the 95^th ^percentile of the other moisture indicators (except the sum of *Mucorales *and species of the indicator genera) lay at 10,000 cfu/g (usually rounded up, *Stachybotrys chartarum *at 3,000 cfu/g, only one sample with *Stachybotrys*). The indirect cultivation method yields statistically very uncertain values for plate colonization (total count) under 10 cfu/plate (< 10,000 cfu/g) and incidental findings below 4 cfu/plate [2,3].

This is confirmed by Schleibinger et al. [6], who found among 19 genera a significantly more frequent occurrence only of *Aspergillus sp*., *Penicillium sp*. and *Eurotium sp*. in dwellings with visible mould contamination (n = 43). As in our previous analysis, this result shows that a massive increase of other moisture indicators is only rarely observed in cases of moisture damage (see 1 case with *Wallemia spp*. in [1]). Recently, it was shown that the indicator genera were associated with the extent of moisture damage in a house [11]. Typical hydrophilic fungi such as *Stachybotrys sp*. are found only under very wet conditions. Growth of mould occurs much sooner at more arid conditions, as some xerophilic species can grow even at 70% water activity upwards. The absence of typical other moisture indicators (in this case, particularly the hydrophilic species) in background measurements in dry dwellings was also confirmed by Horner et al [12].

In summer, three cases out of all background measurements (n = 157) exceeded the additional moisture assessment parameters [9] for the three highest total counts measured (case 1: total cfu 4,100,000/g, 7 fungal moisture indicators; case 2: total cfu 5,100,000/g, 5 fungal moisture indicators; case 3: 9,400,000 cfu/g, 6 fungal moisture indicators). This supports the concept that species other than those of the indicator genera are carried indoors by the outdoor air, depending on the total count, e.g. seasonal influence during summer.

### Mass or surface relation

The background measurements of the UFOPLAN study [9] were intended to answer the question of whether the reference standard of house-dust samples should be the mass of house dust [1,5,6,9,10] or the vacuumed area. Measuring mould load with reference to an area (cfu/m^2^) would not have been expedient. In this case, the 3 examined regions would not have provided comparable results (differences in the collected dust amounts per m^2 ^by factors of 2 to 4). The reason for the failure of the reference area are chiefly the non-reproducible suction conditions (different suction power of pumps, different suction power during sampling caused by the increasing filter resistance, variable frequency of aspiration-head strokes despite a set time interval).

### Sampling with filter holder or with vacuum-cleaner

To collect dust, the Vampirette vacuum-cleaner (Siemens, Germany) [5] or a filter holder [9,13] with a polycarbonate filter (diameter 5 cm) was used. All accessible areas [5] or 2 m^2 ^of floor space [9,13] of a room were vacuumed within 10 minutes. Dust samples were sieved at 63-μm using either a sieving machine [9,13] or manually [5]. Proof that the two practiced sampling and processing techniques (vacuum cleaner, hand sieve vs. filter holder, sieving machine) are equivalent in terms of the indicator genera, and that averaging 4 sampling areas of 0.5 m^2 ^each is sufficient to obtain representative results, is provided by the confirmation of the concentration limits by Baudisch et al. [5] for the main criterion (1^st ^condition or main criterion for moisture damage = exceeding the total count of 500,000 cfu/g) and the indicator genus *Aspergillus spp*. and *Eurotium spp*. (20%) in the total-colony count range of 500,000 to 2,000,000 cfu/g (2^nd ^condition = exceeding at least one secondary criterion for the indicator genera) provided by the measurements with the filter holder in uncontaminated homes. Previously, a reference measurement with vacuum-cleaner and filter holder ([1] table 2, cases 9a und b) also yielded corroborating results.

When purchasing a new filter holder, a large aspiration head is recommended (∅ 10 mm, flow rate 42 l/min is better than ∅ 6 mm and flow rate 15 l/min), in order to collect sufficient quantities of house dust, especially in well-kept homes.

From a logical point of view, in rooms with moisture damage, "false negative" findings can occur when antifungal paint is used (one case with no visible mould colonization, average indoor humidity 80%) or with under-floor heating in winter (one case with affected wall area of 600 cm^2^). "False positive" findings also were observed in cases of reverse airflow from germ-infested ventilator systems [5].

## Conclusion

The major errors during the quantitative evaluation of the presence of mould fungi in house dust samples result from missing filtering to 63 μm, contamination with old dust and the high influence of soil. If the assessment is guided by indicator genera, the percentage standard deviation lies in a moderate range. Measuring mould load with reference to an area (cfu/m^2^) would not have been expedient. Different methods of sampling with vacuum cleaner or the filter holder proved to be equivalent. A temporary storage of the samples before cultivation up to one week appears possible.

## Competing interests

The authors declare that they have no competing interests.

## Authors' contributions

CB and AK conceived the study. CB and AK designed and coordinated the study, CB, OA, and AK analyzed the data and wrote the first draft of the manuscript. OA finalized the manuscript. All authors helped to draft the manuscript and read and approved it in its final form.
